# Introduction to supported employment

**DOI:** 10.1017/S2045796020000979

**Published:** 2020-11-18

**Authors:** Robert E. Drake

**Affiliations:** Westat, Hanover, New Hampshire, USA

Over the last five decades, treatment approaches to serious mental illness have changed substantially. Beginning in the 1960s and 1970s, political, economic, legal, ethical, and medical forces converged to reduce long-term hospitalisation, a movement that has progressed steadily in most countries (Metcalfe and Drake, 2020). As a result of deinstitutionalisation, researchers recognised that much of the passivity, cognitive deterioration, and erosion of social motivation, which had been ascribed to illness, were due to the stagnant environment and hopeless expectations in large hospitals (Rosenberg, 1970). In other words, patients had been socialised into disability. Although funding and implementation of deinstitutionalisation were inadequate in most settings, many people released from long-term institutional care did well and demonstrated renewed motivation (Mechanic, 1985).

Deinstitutionalisation produced several findings in the 1980s. Without the experience of long-stays in the hospital, young people with serious mental disorders presented a different set of challenges. Living in the community, they tried to keep up with peers and sought normative activities, but their symptoms interfered with development, and they often drifted to using substances with peers who were also outside of mainstream school and employment (Caton *et al*., 1989). Both the older, deinstitutionalised patients and the young, never institutionalised patients rejected standard community treatment plans involving clinic visits, medication compliance, case management, and segregated settings such as group homes and day centres. A new approach, called assertive community treatment, emphasised outreach, teaching skills in the community, and greater community integration. It was demonstrably more effective in helping people with serious mental illness adjust to living in the community and avoid hospitalisations (Stein and Test, 1980). In parallel, a new focus on early intervention developed in Australia, tailoring services to the individual needs of young patients early in the course of psychosis and showing better outcomes (McGorry, 2015).

In the 1990s, researchers developed and validated a number of evidence-based practices in community settings (Drake *et al*., 2001). In addition, peer organisations, advocates, and professionals began to promote the idea that people with serious mental illness wanted more than maintaining community tenure; instead, they sought to move beyond controlling illness to develop an active, meaningful life in the community – a movement that became known as ‘recovery’ (Anthony, 1993). Recovery implied living, learning, working, socialising, and participating fully in community life rather than being consigned to segregated settings and dependent roles. Recovery-oriented services, such as supported housing, supported employment, and supported socialisation, began to appear, and rigorous research validated the concepts (Drake *et al*., 1996; Tsemberis *et al*., 2004).

However, despite great hopes, the early 2000s revealed that society and government lacked the political will to support the recovery movement. Even in high-income countries, only a minority of people with serious mental illness were able to access evidence-based services, usually after long delays (OECD, 2014). In the U.S., where recovery ideology and related research flourished, funding for services did not follow. Instead, hospitals, professionals, the pharmaceutical industry, and insurance companies continued to control the package of available services. Thus, rather than recovery-oriented services, people with serious mental illness experienced polypharmacy, housing deficits, unemployment, and stigma. Even worse, the lack of basic social services produced homelessness, a backlash against community-based care, and a new wave of institutionalisation in jails and prisons. When the great recession occurred in 2008, state mental health budget cuts exacerbated problems. Many community mental health centres did not survive, and those that did had to rely on insurance funding, which excluded important social and psychological interventions. The U.S. demonstrated, once again, that medical solutions for social problems are expensive and ineffective. Outside the U.S., many high-income countries with more public health orientation fared better, but none implemented a full recovery-orientation (Slade and Longden, 2015).

The 2010s have been in many ways worse, even before the COVID pandemic, due to the decline of liberal democracy, disparities in wealth, and the increased discrimination against minorities occurring over much of Europe and America (Mounk, 2018). Calls for increasing hospital-based care are now common, following increases in homelessness and criminalisation of mental illness, and polypharmacy persists despite evidence that it produces more harm than benefit. Nevertheless, some positive signals have emerged. In the U.S., these include efforts to improve and decrease police participation in crisis services (SAMHSA, 2020), acceptance that several cycles of pharmacological breakthroughs have failed (Harrington, 2019), and a new focus on social determinants of mental health (Sederer, 2016).

Perhaps the most promising recent development has been the steady adoption of individual placement and support (IPS supported employment) worldwide, based on the notion that employment is a health intervention – the topics of the two following essays. According to these essays, countries throughout the OECD now recognise that employment leads to improved health, perhaps even more for people with disabilities than for the general public. Bond *et al*. (2020) assert that ‘The widespread expansion of IPS has not occurred by chance. IPS is a well-defined, easily understood innovation, which responds to the employment aspirations of service users and families.’ Drake and Wallach (2020) argue that ‘Unlike most mental health treatments, employment engenders self-reliance and leads to other valued outcomes, including self-confidence, the respect of others, personal income, and community integration.’ Both essays agree that governments should do whatever is possible to maximise employment opportunities for people with disabilities, especially those with mental disorders who have traditionally had extremely low rates of integrated, competitive employment. My own experience over five decades of research on mental health interventions suggests that supported employment may be the most effective intervention we have and deserves greater dissemination in many countries.
